# The affinity of the FimH fimbrial adhesin is receptor-driven and quasi-independent of *Escherichia coli* pathotypes

**DOI:** 10.1111/j.1365-2958.2006.05352.x

**Published:** 2006-08-23

**Authors:** Julie Bouckaert, Jenny Mackenzie, José L de Paz, Beatrice Chipwaza, Devapriya Choudhury, Anton Zavialov, Karin Mannerstedt, Jennifer Anderson, Denis Piérard, Lode Wyns, Peter H Seeberger, Stefan Oscarson, Henri De Greve, Stefan D Knight

**Affiliations:** 1 Department of Ultrastructure, Vrije Universiteit Brussel, Flanders Interuniversity Institute for Biotechnology (VIB) Pleinlaan 2, 1050 Brussels, Belgium; 2 Department of Molecular Biology, Swedish University of Agricultural Sciences, Uppsala Biomedical Center PO Box 590, SE-751 24 Uppsala, Sweden; 3 Laboratory for Organic Chemistry, Swiss Federal Institute of Technology (ETH) Wolfgang-Pauli-Str. 10, HCI F315, 8093 Zurich, Switzerland; 4 Department of Organic Chemistry, Arrhenius Laboratory, Stockholm University SE-10691 Stockholm, Sweden; 5 MedImmune 35 W. Watkins Mill Road, Gaithersburg, MD 20878, USA; 6 Department of Microbiology, Academisch Ziekenhuis-Vrije Universiteit Brussel Laarbeeklaan 101, 1090 Brussels, Belgium

## Abstract

Type-1 fimbriae are important virulence factors for the establishment of *Escherichia coli* urinary tract infections. Bacterial adhesion to the high-mannosylated uroplakin Ia glycoprotein receptors of bladder epithelium is mediated by the FimH adhesin. Previous studies have attributed differences in mannose-sensitive adhesion phenotypes between faecal and uropathogenic *E. coli* to sequence variation in the FimH receptor-binding domain. We find that FimH variants from uropathogenic, faecal and enterohaemorrhagic isolates express the same specificities and affinities for high-mannose structures. The only exceptions are FimHs from O157 strains that carry a mutation (Asn135Lys) in the mannose-binding pocket that abolishes all binding. A high-mannose microarray shows that all substructures are bound by FimH and that the largest oligomannose is not necessarily the best binder. Affinity measurements demonstrate a strong preference towards oligomannosides exposing Manα1-3Man at their non-reducing end. Binding is further enhanced by the β1-4-linkage to GlcNAc, where binding is 100-fold better than that of α-d-mannose. Manα1-3Manβ1-4GlcNAc, a major oligosaccharide present in the urine of α-mannosidosis patients, thus constitutes a well-defined FimH epitope. Differences in affinities for high-mannose structures are at least 10-fold larger than differences in numbers of adherent bacteria between faecal and uropathogenic strains. Our results imply that the carbohydrate expression profile of targeted host tissues and of natural inhibitors in urine, such as Tamm-Horsfall protein, are stronger determinants of adhesion than FimH variation.

## Introduction

Urinary tract infections (UTI) occur frequently in humans and are most prevalent in women, who stand an almost 50% chance to experience a UTI in their lifetime. Uropathogenic *Escherichia coli* (UPEC) is the aetiologic agent in about 80% of the reported cases. Acute UTIs can be effectively treated with antibiotics, but chronic recurrence is a problem (Justice *et al*., 2004) as is the emergence of antibiotic-resistant strains (Blahna *et al*., 2006).

Uropathogenic *E. coli* expresses a number of adhesins for specific attachment to carbohydrate-containing receptors on the epithelium of the urinary tract (Berglund and Knight, 2003; Westerlund-Wikström and Korhonen, 2005). This diversity of adhesins allows UPEC to exploit the differential expression of cell surface receptors in distinct parts of the urinary tract, thus generating different clinical outcomes. For example, P-piliated UPEC causes pyelonephritis by binding to galabiose-containingreceptors in the kidney epithelium, while mannose-binding type-1 pili promote cystitis by targeting uroplakin Ia (UPIa) on the mucosal surface of the urinary bladder.

Type-1 pili are important UPEC virulence factors (Mulvey, 2002; Justice *et al*., 2004; Kau *et al*., 2005). They consist of a cylindrical rod of repeating immunoglobulin-like (Ig-like) FimA subunits, followed by a short and stubby tip fibrillum. Pilus assembly occurs through the chaperone/usher pathway. With the help of a ‘donor strand exchange’ mechanism, the immunoglobulin fold of each pilin becomes completed by the amino-terminal extension from the next pilin deposited at the outer membrane usher by the periplasmic chaperone (Zavialov *et al*., 2003; Sauer *et al*., 2004). The FimH adhesin caps the fibrillum of type-1 pili, with a receptor-binding domain (residues 1-158) joined to a pilin domain (residues 159-279) that links the adhesin to FimG, which connects to the rest of the pilus.

The FimH adhesin is responsible for mannose-sensitive bacterial adhesion. The primary physiological receptor for FimH in the urinary tract is UPIa (Zhou *et al*., 2001; Xie *et al*., 2006), a high-mannose glycoprotein abundantly present on the superficial umbrella cells of the uroepithelium. FimH can however, recognize a wide range of glycoproteins carrying one or more *N*-linked high-mannose structures. FimH can also bind yeast mannans and mediate agglutination of yeast and red blood cells. FimH-mediated binding can be inhibited by d-mannose and a variety of natural and synthetic saccharides containing terminal mannose residues (Firon *et al*., 1983; 1984; Sharon *et al*., 1983; Neeser *et al*., 1986; Rockendorf *et al*., 2002). This apparently broad binding range is based on the structural requirement of FimH for an α-linked mannose, either in free form or at the non-reducing end of a glycan (Hung *et al*., 2002).

Despite the high conservation (> 98%) of *fimH* alleles from different *E. coli* isolates (Abraham *et al*., 1988; Hung *et al*., 2002; Vandemaele *et al*., 2003), minor sequence differences have been indicated to correlate with differential binding and adhesion phenotypes (Sokurenko *et al*., 1994; 1995; 1997; 1998). Intriguingly, most of the variable residues are located far away from the mannose-binding pocket and are instead close to the linker region between the receptor-binding domain and the pilin domain. The mannose-binding pocket is highly conserved across all known FimHs, except for an asparagine-to-lysine mutation at position 135 in the FimH from genome sequenced O157:H7 enterohaemorrhagic *E. coli* (EHEC). This mutation has been predicted to abolish mannose binding (Hung *et al*., 2002). O157:H7 is the best characterized EHEC serotype and the causative agent of ‘hamburger disease’.

The fine specificity of FimH had not been characterized yet in molecular detail, despite considerable interest in understanding the basis for FimH-mediated adhesion. This interest originates from long-standing observations that blocking the FimH-receptor interaction prevents bacterial infection (Aronson *et al*., 1979; Langermann *et al*., 1997; Sharon, 2006). We investigated, in molecular detail, the binding properties of FimH receptor-binding domains from the *E. coli* laboratory strain K-12, the J96 and CI#4 UPEC strains, the intestinal isolate F-18 *E. coli* as well as four EHEC strains. The fine specificity of FimH for high-mannose epitopes was probed using a series of oligomannosides corresponding to substructures of high-mannose *N*-linked glycans on proteins.

## Results

### Binding of uropathogenic FimH to a high-mannose microarray

**Fig. 1:**
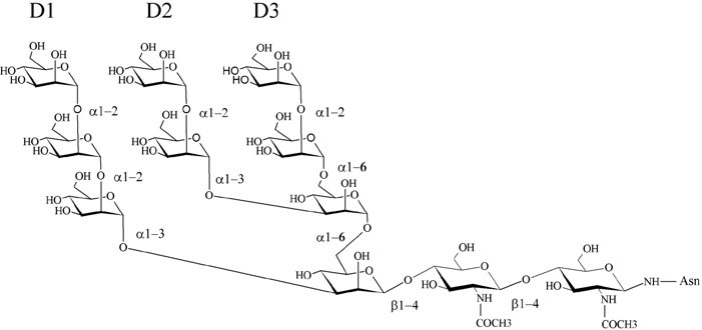
Structure of the largest high-mannose glycan, oligomannose 9, indicating the D1, D2 and D3 arms.

**Fig. 2:**
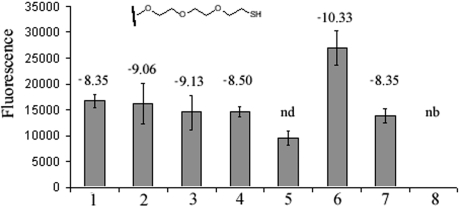
Fluorescence signal observed for high-mannose microarray binding by FimHrb_J96_ at 0.5 mM. The immobilized oligosaccharides are linked at their reducing end to a thiol-terminated triethylene glycol (insert). Compounds 1-3 are oligomannose 9, 6 and 3, respectively, minus the chitobiose (GlcNAc)_2_ (Fig. S1), β-linked like in high-mannose structures (Fig. 1). Manα1-2Manα1-2Man (4), Manα1-2Manα1-6Manα1-6Man (5), d-mannose (6) and Manα1-6Manα1-6Man (7) are all α-linked on the array (Fig. S1), as in natural high-mannose (Fig. 1). d-galactose (8) is β-linked (Fig. S1). Numbers above the bars are ΔG values (kcal mole^−1^), calculated from the affinities measured by SPR (Table 2) for identical (4, 6 and 7) or similar (1-3) compounds. nb, no binding; nd, not determined.

A high-mannose microarray (Adams *et al*., 2004; Ratner *et al*., 2004) was employed to study the carbohydrate binding specificity of FimH from UPEC strain J96. For this purpose, a series of high-mannose oligosaccharides was synthesized (Fig. S1) (Ratner *et al*., 2002), including several substructures of the triantennary oligomannose 9 (Fig. 1). The synthetic oligosaccharides were linked onto the array in the anomeric configuration in which they occur in *N*-linked high-mannose structures (Fig. S1), via a thiol-terminated triethylene glycol linker that permits their attachment onto maleimide-functionalized glass slides through the formation of a stable covalent bond. A standard DNA array printer was used to create a carbohydrate microarray of 480 spots (15 replica per sample; Fig. S2). The use of carbohydrate solutions with different concentrations, ranging from 2 mM to 0.25 mM, allowed the estimation of the carbohydrate binding profile for the receptor-binding domain of FimH_J96_, FimHrb_J96_, by comparing the fluorescence intensities of spots at subsaturating concentration levels. The results unambiguously showed that FimHrb_J96_ bound to all mannosides (Figs. 2 and S2) and, with the exception of d-mannose, gave rise to similar fluorescence signals. No signal was observed for β-linked d-galactose, which served as the negative control.

### Binding of linear trimannosides to FimH from uropathogenic and faecal *E. coli* strains

**Fig. 3:**
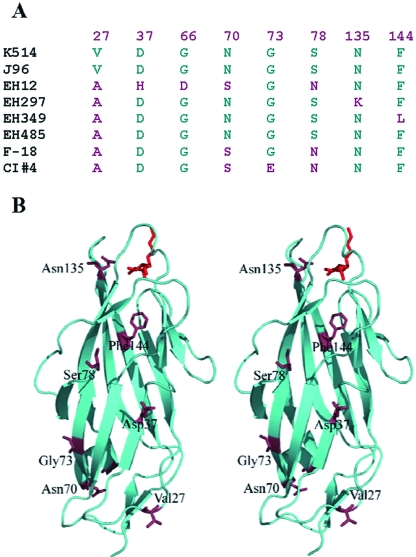
Sequence (A) and location (B) of the variations (raspberry red) in the FimH receptor-binding domains of studied *E. coli* strains. A bound butyl α-d-mannoside (red ball-and-stick model) indicates the location of the binding site (Bouckaert *et al*., 2005).

**Fig. 4:**
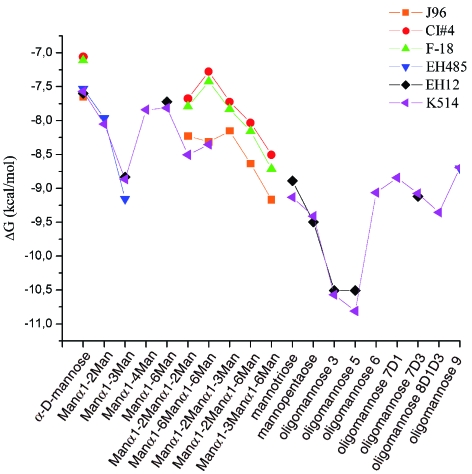
Comparison of the binding profiles of FimH variants with high-mannose substructures. The Gibbs free energy of binding was calculated from the measured affinities of FimHrb_J96_, FimHrb_F-18_ and FimHrb_CI#4_ for the trimannosides used for displacement of [^3^H]d-mannose (Table 1), and of FimHrb_EH485_, FimHrb_EH12_ and FimHrb_K514_ for the oligomannosides used in the SPR assay (Table 2). The *x*-axis displays their common mannoside moieties.

To investigate if allelic differences in *fimH* cause differences in carbohydrate binding at the molecular level, mannoside binding of the FimH receptor-binding domains from a faecal F-18 (FimHrb_F-18_) and a uropathogenic CI#4 (FimHrb_*CI*#*4*_) *E. coli* isolate were compared with the previously characterized FimH receptor-binding domain from the uropathogenic *E. coli* J96 strain (FimHrb_J96_), using the [^3^H]d-mannose displacement assay (Table 1) (Bouckaert *et al*., 2005). F-18 and CI#4 isolates, as well as an isogenic strain expressing *fimH*_F-18_ and *fimH*_*CI*#*4*_, have previously been shown to mediate distinct adhesion patterns (Sokurenko *et al*., 1995). FimHrb_F-18_ and FimHrb_CI__#4_ differ from FimHrb_J96_ by substitutions Val27Ala, Asn70Ser and Ser78Asn (Fig. 3A). In addition, FimHrb_CI__#4_ has a Gly73Glu substitution. None of these residues are located close to the mannose-binding pocket (Fig. 3B). The equilibrium dissociation constants of FimHrb_J96_, FimHrb_F18_ and FimHrb_CI__#4_ for five trimannosides, which correspond to linear substructures of high-mannose glycans (Fig. 1), are in the range of 0.35-7.5 μM (Table 1). Remarkably, a similar binding profile is observed for all three FimH variants (Fig. 4).

**Table 1:** Binding of linear trimannosides to FimH from three different *E. coli* strains.

	K_*d*_ (nM) (at 37°C)		
	
Ligand	J96	CI#4	F-18
d-mannose	4100	10 700	9800
Manα1-2Manα1-2ManαOMe	1600	3 950	3250
Manα1-2Manα1-3ManαOMe	1800	3 650	3050
Manα1-2Manα1-6ManαOMe	830	2 200	1800
Manα1-3Manα1-6ManαOMe	350	1 030	730
Manα1-6Manα1-6ManαOMe	1400	7 500	5900

Affinity measurements via the displacement of [^3^H]d-mannose. The trimannosides correspond to the branches of the high-mannose tree (Fig. 1) and are methylated at their reducing end.

**Table 2:** K_*d*_ as measured by surface plasmon resonance.

	K_*d*_ (nM) (at 25°C)				
	
Mannose and linear mannosides (Compound numbers on glycan microarray for ΔG calculation)	EH297	EH349	EH485	EH12	K514
*E. coli* strain					
d-mannose	NB	2620	3030	2700	2830
Manα-triethylene glycol (6)			ND	ND	27
Manα1-2Man			1460	ND	1260
Manα1-3Man			196	338	320
Manα1-4Man			ND	ND	1800
Manα1-6Man			ND	2190	1880
Manα1-2Manα1-2Manα-triethylene glycol (4)			ND	ND	587
Manα1-6Manα1-6Manα-triethylene glycol (7)			ND	ND	756

Branched mannosides		EH485	EH12	K514
Mannotriose (3)	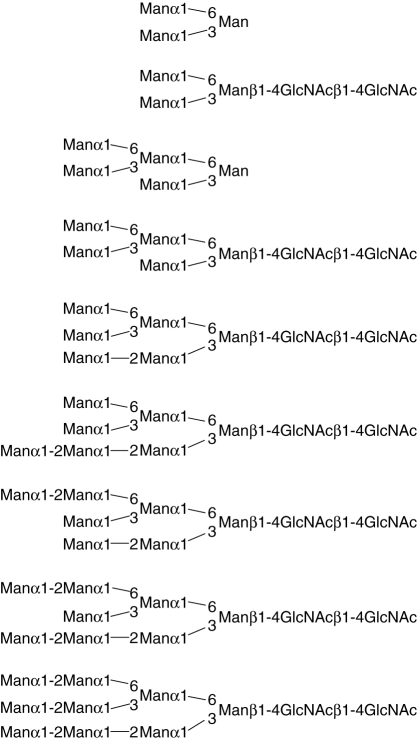	ND	307	204
Oligomannose 3		ND	20	18
Mannopentaose		ND	110	127
Oligomannose 5		ND	20	12
Oligomannose 6 (2)		ND	ND	229
Oligomannose 7D1		ND	ND	332
Oligomannose 7D3		ND	208	224
Oligomannose 8D1D3		ND	ND	140
Oligomannose 9 (1)		419	ND	426

NB, no binding; ND, not determined.

### Docking of linear trimannosides to FimH

**Fig. 5:**
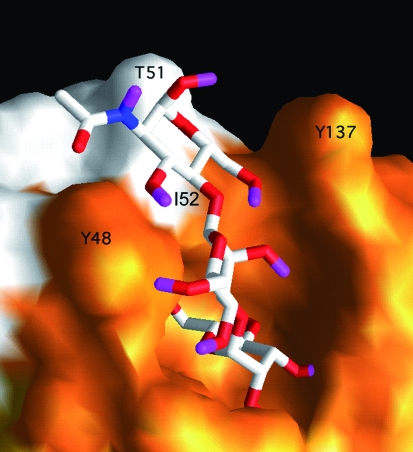
Predicted (AutoDock3) mode of binding of Manα1-3Manβ1-4GlcNAc (ball-and-stick representation) to FimH (PDB entry code 1TR7) [molecular surface presentation using GRASS (Nayal *et al*., 1999)]. The figure illustrates how the non-reducing mannose is buried in the cavity and the middle mannose and GlcNAc at the reducing end insert in the tyrosine gate between Tyr48 and Tyr137.

AutoDock3 was used to carry out docking studies to the FimH_J96_ mannose-binding site for six linear trisaccharides that correspond to substructures of high-mannose: the trimannosides from Table 1 and Manα1-3Manβ1-4GlcNAc. Docking produced one predominant binding mode, whereby the mannose residue at the non-reducing end is inserted in the mannose-binding pocket in the same way as experimentally observed for d-mannose in the FimH-mannose crystal structure (Bouckaert *et al*., 2005). The best docked energy is predicted for Manα1-3Manβ1-4GlcNAc (Fig. 5). Its middle mannose residue as well as the *N*-acetylglucosamine residue at the reducing end are predicted to pack against the tyrosine gate, shaped by Ile52, Tyr48 and Tyr137. Also Thr51 interacts, previously shown to hydrogen bond with the carbonyl group on methyl umbelliferyl mannose (Bouckaert *et al*., 2005).

### FimH variation in enterohaemorrhagic *E. coli* strains

To obtain an overview of the range of variation in FimH from EHEC strains, FimH from 22 EHEC isolates were sequenced (Fig. S3). A selection was made from the 22 new sequences of EHEC FimH, which best reflects the observed spectrum of variations in FimH, in an effort to assess the contributions of multiple, concurrent variant residues in the FimH receptor-binding domain to differences in FimH affinity and to bacterial adhesion. FimH receptor-binding domains from four EHEC variants were produced and used for binding studies (Table 2). FimHrb_K514_, originating from *E. coli* strain K514 and with the same sequence as the UPEC FimHrb_J96_, was used as the reference FimH. FimH_EH12_ originates from serotype O2:K1:H6, whereas FimH_EH485_, FimH_EH349_ and FimH_EH297_ originate from O157:H7 *E. coli* strains. The FimH sequence variation in EHEC involves largely the same residues as in faecal and uropathogenic *E. coli* (Fig. 3A), except for the Asn135Lys mutation. FimHrb_EH485_ differs from FimHrb_J96_ or FimH_K514_ at residue 27 only, which is an alanine as in all 22 sequenced EHEC FimH proteins. FimHrb_EH297_ in addition has the Asn135Lys change that has been predicted to abolish mannose binding (Hung *et al*., 2002), whereas FimHrb_EH349_ has Phe instead of Leu at position 144, which is located directly underneath the mannose-binding pocket. FimHrb_EH12_ has the Asn70Ser and Ser78Asn substitutions, typical for *fimH* alleles from faecal *E. coli* isolates, as well as two rare substitutions (Asp37His and Gly66Asp) (Fig. 3). Because its sequence is the most different and has some of the common faecal alleles, FimHrb_EH12_ was most frequently selected for extensive comparison of oligomannoside affinities with FimHrb_K514_ (Table 2).

### Oligomannoside binding to FimH reveals strain-independent binding profiles

The solution equilibrium binding constants of FimH for most natural oligomannoside substructures, present in the glycan part of high-mannose glycoproteins (Fig. 1), were characterized in an surface plasmon resonance (SPR) competition experiment (Table 2). We observe essentially the same glycan affinities for FimHrb_K514_ and the FimH receptor-binding domains from EHEC strains (Table 2, Fig. 4). Two of the linear trimannosides used in the [^3^H]d-mannose displacement experiments (Table 1), modified to have triethylene glycol instead of a methyl group at their reducing end, were also included in the SPR measurements (Table 2). As shown previously (Bouckaert *et al*., 2005), there is a good agreement in binding constants obtained using either of the two techniques (SPR or [^3^H]d-mannose displacement). Most importantly, we observe nearly the same affinities among UPEC and EHEC FimH and alike binding profiles for all studied FimH variants (Fig. 4).

### FimH variation has a limited but clear effect on haemagglutination

To gain insight into the basis of differential bacterial adhesion caused by FimH variation, we compared the haemagglutination titres of the isogenic strains, complemented with recombinant variant or mutant FimH, and we also measured the haemagglutination of the wild-type isolates (Table 3). An isogenic, *fimH*-negative strain was complemented with recombinant *fimH* variants and mutants. No large differences were observed in haemagglutination titres between the *fimH*-complemented isogenic strains, except for *fimH*_EH297_ that was negative. The recombinant strain carrying the single Ser78Asn mutation in FimH gives the same agglutination titre as those with *fimH*_K514_ (K-12 *E. coli*) and *fimH*_J96_ (UPEC). The isogenic strains producing FimH mutants Asn70Ser and Asn70Ser/Ser78Asn agglutinated twofold less. The interest for these mutations comes from the frequent occurrence of the double FimH mutant Asn70Ser/Ser78Asn, as in the EH12, F-18 and CI#4 strains of our study (Fig. 3). The recombinant strains expressing variants *fimH*_EH12_, *fimH*_EH349_ and *fimH*_EH485_ led to twofold, two- to fourfold and four- to eightfold less haemagglutination respectively. The wild-type O157:H7 isolates EH349, EH297 and EH485 did not haemagglutinate at all, whereas the non-O157 EH12 isolate and K514 agglutinate to the same titre as the isogenic strain carrying *fimH*_J96_. In summary, the original strains, corresponding to those *fimH* variants that perform least in their recombinant form in the isogenic strain, fail to haemagglutinate (Table 3).

**Table 3:** Haemagglutination titres of *E. coli* expressing *fimH* alleles.

*fimH*	Pathotype	Isolates	Recombinant
J96	UPEC	1	1
K514	K-12	1	1
EH12	EHEC	1	1/2
EH349	EHEC	0	1/2-1/4
EH485	EHEC	0	1/4-1/8
EH297	EHEC	0	0
S78N	Mutant	NA	1
N70S	Mutant	NA	1/2
N70S, S78N	Mutant	NA	1/2

NA, not applicable.

### The Asn135Lys mutation renders O157:H7 FimH inactive

Another important observation is that FimHrb_EH297_ does not bind any mannosides (Table 2). EH297 is an O157:H7 isolate that carries the Asn135Lys mutation in the mannose-binding pocket. FimHrb_EH297_ does not recognize the monoclonal antibody 1C10 Fab that is used in the SPR binding assay. This is consistent with 1C10 being competitive with mannose for binding of the FimH mannose-binding pocket. Moreover, neither the wild-type EH297 isolate, nor the isogenic strain that has been complemented with full-length FimH_EH297_, haemagglutinate red blood cells. These data give for the first time proof that the Asn135Lys mutation renders FimH inactive for mannose-receptor binding. Among the 22 new sequences of *fimH* from EHEC strains (Fig. S3), all non-O157 strains carry FimH with an asparagine at position 135. On the other hand, only five of 11 O157 strains carry FimH with a functional mannose-binding pocket. Non-O157 EHEC as well as some O157 could thus be implicated in non-diarrhoeal haemolytic uraemic syndrome (HUS) associated with UTIs (Starr *et al*., 1998; Hogan *et al*., 2001).

## Discussion

A large body of data on FimH-mediated adhesion has accumulated over the years from studies investigating the binding of whole piliated bacteria to yeast cells, yeast mannan, red blood cells, fibronectin and derived peptides (Sokurenko *et al*., 1994; 1995), collagen (Pouttu *et al*., 1999), urothelial or bladder and buccal or gut cell lines (Sokurenko *et al*., 1998; Duncan *et al*., 2005). Whereas such studies are useful for understanding bacterial adhesion at a cellular level, the availability of soluble FimH truncates containing the receptor-binding domain allowed us to characterize FimH receptor recognition at the molecular level. The ratios of the affinities of FimH that we measured in the current study for different oligomannosides correlate remarkably well with the ratios of oligomannoside concentrations needed for 50% inhibition of bacterial adhesion (Firon *et al*., 1982; 1984). A link between cellular and molecular FimH-receptor binding can thus be established.

Mannose binds to all FimH variants examined with a dissociation constant in the micromolar range (Tables 1 and 2), corresponding to unusually tight lectin binding of a monosaccharide. The high affinity is explained by the structures of the FimH receptor-binding domain in complex with mannose, which reveals a deep binding pocket that almost completely buries the sugar and interacts strongly with all of its hydroxyl groups except for the anomeric one (Hung *et al*., 2002; Bouckaert *et al*., 2005). All mannosides tested in the high-mannose microarray experiment bound to FimH and gave rise to alike fluorescence signals (Figs. 2 and S2), with the single exception of d-mannose, which bound significantly better. The strong signal for FimH binding to d-mannose on the array is in agreement with the enhanced affinity of α-d-mannose triethylene glycol over d-mannose as measured by SPR (Table 2). The direct linkage of mannose to triethylene glycol (Fig. 2, insert) at the reducing end of mannose appears to substantially increase the affinity of mannose for FimH, through interactions with the tyrosine gate, similar to what has been found for aliphatic linkers on mannose (Bouckaert *et al*., 2005). Di- and oligomannosides do not undergo a similar enhancement in affinity, as the distance between the FimH-binding non-reducing mannose and the linker becomes too large. The resulting high fluorescence signals should be considered an artefact if one only takes the attached carbohydrate into consideration; however, they represent true signals that reflect the recognition of the mannose inclusive its linker. This demonstrates the potential impact of linkers on (mono)saccharides used to attach the carbohydrate to microarrays or sensor chips and also shows that large differences in binding strength can be distinguished using microarrays. Hence we are particularly encouraged by the potential of the carbohydrate microarray technology for high-throughput screening of inhibitors to prevent FimH-mannose interactions. Compound 3, or mannotriose, does not give an outstanding signal for FimH binding on the microarray. It differs from oligomannose 3 by the lack of a chitobiose core (GlcNAc)_2_ (Table 2). Presence of the chitobiose unit is expected to elevate the fluorescent signal towards a level similar as for α-d-mannose triethylene glycol (Fig. 4 and Table 2). Comparison with ΔG values (Fig. 2) clarifies that whereas a distinction can be made for large differences in affinity using the glycan microarray, quantification of the results for closely related affinities is difficult.

Type-1 fimbriated bacteria from different *E. coli* clinical isolates have altered mannoside binding and adhesion profiles. Recombinant *E. coli* expressing a CI#4 (UPEC) FimH variant adhere tightly to yeast mannan, A498 human kidney cells, and J82 human bladder cells, whereas bacteria expressing FimH from the faecal *E. coli* F-18 strain show poor adhesion to all three substrates (Sokurenko *et al*., 1997). Minor sequence variations in FimH have been held responsible for these differences (Sokurenko *et al*., 1994; 1995; 1997; 1998). However, we do not observe a correlation between FimH variation and the affinities or specificities of the variant FimH receptor-binding domains for oligomannosides (Tables 1 and 2). As an example, our data show that there are no significant differences between faecal FimHrb_F-18_ and UPEC FimHrb_CI#4_ in their affinities for d-mannose or trimannoside (Table 1). Hence, the single Gly73Glu amino acid difference between these two FimH variants does not directly impact affinity. This suggests that factors other than differences in mannose binding of FimH *per se* cause the different adhesion phenotypes.

Many of the variable residues of FimH are located near the base of the receptor-binding domain (Fig. 3B), in the vicinity of the flexible linker connecting the receptor-binding and pilin domains in FimH. Based on computer simulations, it has been suggested that minor sequence differences in or near the linker region might affect the ease with which the receptor-binding domain can undergo conformational changes upon bacterial adhesion, as these changes propagate from the linker strand to the mannose-binding site (Thomas *et al*., 2002). Nonetheless, FimH receptor-binding domains rarely exceed 1% difference in identity and the FimH pilin domain is even more conserved. FimA sequences display larger differences in identity (91-99%) than FimH (Vandemaele *et al*., 2003). It was already suggested early on that the fimbrial shaft, which is almost exclusively built from FimA, imposes the presentation of the FimH adhesin on the bacterial surface (Firon *et al*., 1983). The fimbrial shaft has recently been shown as an important determinant in the sugar fine specificity of bacterial adhesion phenotypes (Madison *et al*., 1994; Duncan *et al*., 2005). In conclusion, differences between FimH variants, with the exception of those carrying the Asn135Lys mutation, are observed only when FimH is expressed in the context of its fimbrial shaft, whereas in its soluble form the FimH receptor-binding domains exhibits the same tissue tropism.

The number of fimbriae per cell and the number of fimbriated cells could of course also influence bacterial adhesion. The limited information to date indicate that these numbers are rather constant (McCormick *et al*., 1989); however, it may be difficult to determine numbers of fimbriae with the accuracy required to interpret the relatively small differences in the number of adherent bacteria between strains. The multivalent nature of the bacterium-substratum interaction would further blur small differences in fimbrial numbers, by enhancing its effects on bacterial adhesion in a non-linear manner. In our haemagglutination study, expression of recombinant *fimH*_EH12_, *fimH*_EH349_ or *fimH*_EH485_ in an isogenic strain have a twofold, two- to fourfold and four- to eightfold lower haemagglutination titres compared with recombinant *fimH*_J96_ in the same isogenic strain. This degree of differentiation agrees well with the reported 2-12-fold differences in number of adherent bacteria for isogenic strains expressing *fimH* alleles (Sokurenko *et al*., 1995; 1997). Remarkably, O157:H7 EHEC isolates EH349 and EH485 do not agglutinate rabbit red blood cells and are the parent strains for those FimH variants that haemagglutinate least optimal when expressed in their recombinant form in the isogenic strain (Table 3). Taking into account that all variant receptor-binding domains display similar affinities (Tables 1 and 2; Fig. 4), with the exception of the non-binding FimH_EH297_ variant, FimH variation could possibly affect pilus assembly. The tip adhesin is indeed crucial to initiate pilus assembly by interactions between the FimH receptor-binding domain and the usher outer membrane export channel (Barnhart *et al*., 2003).

All FimH variants have similar mannose binding profiles (Fig. 4), suggesting their binding sites are structurally highly conserved. The differential binding of the individual oligomannoses presumably depends on how well the carbohydrate fits into the tyrosine gate (Bouckaert *et al*., 2005). Both Manα1-3Man and the linear trisaccharide, Manα1-3Manα1-6ManαOMe, bind about 10-fold tighter than d-mannose (Tables 1 and 2). The branched carbohydrates mannotriose and mannopentaose do not bind significantly better than their linear moieties. Instead they bind comparably to Manα1-3Man, making the existence of a second binding site or subsite on FimH unlikely. Affinity for FimH is enhanced by another factor of 10 through β1-4 linkage of the non-reducing Manα1-3Man to the GlcNAc residue in the chitobiose core of *N*-glycosylated proteins. The β-linkage of mannotriose alone does not lead to outstanding fluorescence signals on the microarray (compound 5 in Figs 2, S1 and S2), to match the high affinity of oligomannose 3 with that of triethylene glycol-linked α-d-mannose (Table 2), but rather behaves like oligomannoses 6 and 9. This suggests that GlcNAc in itself, rather than the anomeric state of the glycosidic linkage, accounts for enhanced binding. Our docking studies predict that enhanced binding of Manα1-3Manβ1-4GlcNAc is accomplished by interactions of the central mannose and GlcNAc in the tyrosine gate (Fig. 5).

In those oligomannosides (oligomannoses 3 and 5) where the D1 arm, Manα1-3Manβ1-4GlcNAc, is not hampered at the non-reducing end by an α1-2 linked mannose residue (Rosenstein *et al*., 1988), the affinity is very high (around 20 nM, Table 2), paralleling the affinity of FimH for aryl and alkyl mannosides (Bouckaert *et al*., 2005). Oligomannoses with a substituted D1 arm but with a free D2 arm (oligomannoses 6, 7D1, 7D3 and 8D1D3) reach affinities comparable to those for Manα1-3Man, indicating that FimH largely targets the D2 arm in those cases. The D2 arm is further substituted with an α1-2 linked mannose residue in oligomannose 9. The largest oligomannose is therefore not the best binder. Nevertheless, both the length and the multivalency of the oligomannoses make a minor contribution to affinity. Oligomannose 9 benefits over Manα1-2Man from the enhanced probability to encounter an α1-2 linked mannose. Mannopentaose benefits over mannotriose from the twofold presence of terminal Manα1-3Man. Finally, Manα1-2Manα1-2Man has a larger affinity than Manα1-2Man and Manα1-6Manα1-6Man than Manα1-6Man. The extended length of these glycans may increase the number of interactions of these glycans with the tyrosine gate of FimH.

Our results show that a free, non-reducing Manα1-3Manβ1-4GlcNAc on the D1 branch of high-mannose glycans is a 100-fold stronger FimH epitope than α-d-mannose. This trisaccharide, isolated as the main component in the urine from mannosidosis patients (Norden *et al*., 1973), has previously been found to be the best inhibitor of red blood cell agglutination and yeast aggregation studies in the early work of the group of Sharon (Firon *et al*., 1982; 1983; 1984). Interestingly, UTIs are not described as one of the burdens of mannosidosis patients. The origin of this trisaccharide is not known, but could be Tamm-Horsfall protein [THP, uromodulin; reviewed in the study by Devuyst *et al*. (2005)]. THP is a heavily glycosylated GPI-anchored protein located on the cells lining the thick ascending limb of Henle's loop in the kidney. A specific protease cleaves THP and releases large amounts into the urine (∼50 mg day^−1^ in urine from healthy human individuals), making it the most abundant protein in normal human urine. THP provides a first line of defence against UTIs (Pak *et al*., 2001). THP knockout mice have been shown to be more prone to bladder infection by type-1 fimbriated UPEC (Bates *et al*., 2004; Mo *et al*., 2004). There are seven *N*-glycosylation sites in THP, of which one, Asn251, carries high-mannose carbohydrate chains (van Rooijen *et al*., 1999). In humans, the most abundant glycoform is Man6 (75%), followed by Man7 (17%) and Man5 (8%) (Cavallone *et al*., 2004). Man5 is the only one of these glycoforms that exposes Manα1-3Manβ1-4GlcNAc at the non-reducing end of its high-mannose D1 arm (Table 2). In pigs, the most abundant glycoform is also Man6 (53%), but the Man5 content is much higher (47%) than in human THP. Pig THP binds about threefold better than human THP to type-1 fimbriated *E. coli* (Cavallone *et al*., 2004), consistent with our finding that glycan structures exposing the Manα1-3Manβ1-4GlcNAc epitope provide the tightest FimH binding.

The physiological receptor for FimH in the urinary tract is UPIa (Wu *et al*., 1996; Zhou *et al*., 2001). The glycosylation pattern of human UPIa is not known, but the mouse protein presents very low amounts of oligomannose 6, and similar amounts of oligomannose 7, 8 and 9 (Xie *et al*., 2006). Of those, only oligomannoses 6 and 7D1 carry a free, non-reducing mannotriose (see Table 2). This is congruent with our previous finding that interactions of FimH with mannose, and not with mannotriose, are relevant in the human urinary tract, despite the 10-fold higher affinity of FimH for mannotriose (Hung *et al*., 2002). The highest-affinity epitope for FimH, Manα1-3Manβ1-4GlcNAc, is not free for binding to FimH in any of the recently elucidated glycan structures on mouse UPIa (Xie *et al*., 2006). However, a change in the glycosylation of UPIa or other urothelial surface proteins may alter the host susceptibility to UTIs (Xie *et al*., 2006). There is ample evidence that the carbohydrate expression profile of eukaryotic cells can be subject to variation [for a review, see the study by Durand and Seta (2000)] in certain disease states, but also under normal physiological conditions such as menopause and ageing. Interestingly in this regard, diabetic patients are more prone to UTIs than healthy individuals (Hoepelman *et al*., 2003). Type-1-fimbriated *E. coli* adhere twice as many to urothelial cells of diabetic patients than of healthy individuals, independently from the presence of various substances excreted in the urine, such as albumin, glucose and THP (Geerlings *et al*., 2002). Unfortunately, it is currently unknown whether this can be attributed to altered glycosylation of FimH receptors in the urinary tract. A better understanding of the fundamental relationships between physiological conditions of the host and carbohydrate expression on urothelial cells is thus needed.

The differences in affinities of FimH for mannose structures are an order of magnitude larger than previously reported differences in bacterial adhesion. Affinities of FimH for high-mannose substructures differ 100-fold (Table 2), while reported differences in bacterial adhesion are 2-12-fold (Sokurenko *et al*., 1995; 1997). This implies that the carbohydrate expression profile of the targeted host tissues and of protective molecules such as THP could be more important determinants of FimH-mediated bacterial adhesion. Adhesion differences may thus be receptor- rather than adhesin-based. In summary, we determined the specificities of the type-1 adhesin FimH for its high-mannose receptors and demonstrated the conservation of these specificities in UPEC strains expressing variant FimH adhesins. The conserved nature of FimH carbohydrate recognition suggests that tight-binding FimH ligands will have broad range efficiency in preventing type-1 pilus-mediated adhesion and bacterial infection.

## Experimental procedures

### Preparation of FimH receptor-binding (rb) domains

FimHrb_J96_ was expressed from plasmid pPKL241, FimHrb_CI#4_ from plasmid pMAS146 and FimHrb_F-18_ from plasmid pPKL316 in HB101 (Schembri *et al*., 2000). We selected four variants after sequencing of *fimH* from 22 EHEC strains (Fig. S3) to represent the variation in EHEC FimH. EH12 (O2:K1:H6), EH297 (O157:H7) and EH349 (O157:H7) are human clinical isolates, EH485 (O157:H7) was isolated from a bovine carcass. Constructs for FimHrb_K514_, FimHrb_EH12_, FimHrb_EH297_, FimHrb_EH349_ and FimHrb_EH485_ were prepared using the Gateway vector pDEST14 and transformed into *E. coli* C43 (DE3). In all cases, the *fimH* genes were truncated after Thr158, followed by a 6-histidine tag. The same expression and purification protocol was used for all variants as for FimHrb_J96_ (Bouckaert *et al*., 2005).

### Haemagglutination experiments

The effect of FimH variation and mutation on bacterial adherence was evaluated by means of rabbit red blood cell agglutination on ice, both by using the clinical isolates and wild-type strains. Full-length *fimH* genes from natural variants (K514, EH12, EH297, EH349 and EH485 isolates) were cloned into pDONR221 (Gateway). Mutations (N70S, S78N, N70S/S78N) were introduced in the clone pENT53, carrying the full-length *fimH* gene from strain K514. The mutations were designed to simulate the effect of variations between uropathogenic and faecal *E. coli* strains. Transformation into the AAEC185(pUT2002) strain (Minion *et al*., 1989) complements the pUT2002 plasmid, encoding for the whole *fim* operon except for *fimH*. Bacteria were grown statically for 24-48 h at 37°C in Luria Broth (LB), to reach optimal fimbrial expression. Bacteria were pelleted by centrifugation, washed three times in phosphate buffered saline (PBS) and diluted to an equal dispersity at 600 nm. To a twofold dilution series of the bacteria in round-well microtitre plates, 50 μl of 4% fresh rabbit red blood cells were added. Haemagglutination was read out as positive when the red blood cells did not precipitate in the circular centre of the well, but instead remained homogeneously dispersed in the solution through fimbrial interactions with the bacteria.

### Affinity measurements

#### High-mannose microarray

The high-mannose microarray was prepared as described (Fig. S1 and Adams *et al.*, 2004). For hybridization experiments, FimHrb_J96_ (10 μg) was added to a solution containing 10 mM HEPES, 10 mM MES, 150 mM NaCl, 0.5 mM CaCl_2_ and 0.05% Triton X-100 (pH 7.4). In this study, 80-90 μl of the protein solution was placed on the slide and distributed over the surface of the slide by using hybridization coverslips (HybriSlip, Molecular Probes). After 2 h at room temperature, the slides were washed twice with incubation buffer, twice with distilled water, and then centrifuged for 5 min to ensure dryness. For detection of bound FimH, arrays were incubated with Penta-His Alexa Fluor 555 monoclonal antibody (2 μg in 80-90 μl of incubation buffer; Qiagen AG) for 1 h and then washed and dried as described earlier. Microarrays were scanned by using a standard fluorescence slide scanner (LS400 Tecan). The spots were quantified by using Scan Array Express Software (Perkin Elmer).

#### Binding of linear trimannosides via the displacement of [^3^H]d-mannose.

[^3^H]d-mannose was obtained from Amersham. Linear trimannosides were synthesized and dissolved in double distilled water to give stock solutions of 0.87 M Manα1-2Manα1-2ManαOMe, 0.25 M Manα1-2Manα1-3ManαOMe, 0.27 M Manα1-2Manα1-6ManαOMe, 0.30 M Manα1-3Manα1-6ManαOMe and 0.13 M Manα1-6Manα1-6ManαOMe. The binding experiments were performed in duplicate and as described previously (Bouckaert *et al*., 2005).

#### Competition experiments using SPR.

We used Fab fragments of the monoclonal antibody 1C10, recognizing the mannose-binding site of FimH, to determine the solution affinity of FimH-carbohydrate interactions. The monoclonal antibody 1C10 was produced by a mouse hybridoma cell line at Medimmune and its Fab fragments were prepared and purified. All oligosaccharides were purchased from Dextra Laboratories (UK). The SPR measurements were performed on a Biacore3000™ as described previously (Bouckaert *et al*., 2005). Briefly, Fabs of the monoclonal antibody 1C10 were immobilized via amine coupling at 1000 Resonance Units (1000 pg ligand mm^−2^) on a CM5 sensor chip (*BIAapplications Handbook*, Biacore AB, Uppsala, Sweden). The kinetic constants for binding of FimH to the immobilized antibody were first determined, using different concentrations of FimH in phosphate buffered saline with 0.005% surfactant P20 and 3 mM EDTA, at a flow rate of 20 μl min^−1^ and at 298 K. A Langmuir binding isotherm with a 1:1 stoichiometry was fitted to the data to obtain k_*a*_ and k_*d*_ and the maximal binding R_*max*_. Secondly, the equilibrium dissociation constants K_*d*_ of FimH-saccharide interactions were determined in a competition experiment. Samples containing a fixed concentration of FimH (close to the calculated *K*_*d*_ of the FimH-antibody interaction) were used in combination with a concentration range of the sugar. A Langmuir binding isotherm with a 1:1 stoichiometry was fitted to the data, while keeping k_*a*_ and k_*d*_ and R_*max*_ from the foregoing experiment constant, to obtain the concentrations of bound FimH. Every measurement was repeated at least twice, most often including different protein and sugar batches.

### Computational docking

Computational docking of trimannosides to FimH was carried out using AutoDock3 (Morris *et al*., 1998). Because the protein is represented by a grid of affinity potentials in AutoDock3, the protein conformation is fixed during the docking procedure. Flexibility of ligands is accounted for by allowing rotation around flexible torsion angles. The docked energies are calculated as the sum of the intermolecular interaction energy and the internal energy of the ligand. The search for the best interaction energy was carried out by means of the Lamarckian genetic algorithm. Ligand structures were obtained from the PDB-database and prepared for docking by adding hydrogens and charges with AutoDockTools (Sanner *et al*., 1996). Each simulation consisted of 100 independent runs, with a population size of 100, 50 000 generations, and a maximum of 5 000 000 energy evaluations. The large parameter values used here were required to achieve convergence when docking the big and flexible trisaccharide ligands. Solutions were ranked based on their docking energies, and similar solutions were clustered (cluster cut-off r.m.s.d. < 1 Å). The top solutions were visually inspected using O (Jones *et al*., 1991).

## Supplementary material

The following supplementary material is available for this article online:

Fig. S1. High-mannose microarray.

Fig. S2. Fluorescence response for FimH binding to the carbohydrates linked on the high-mannose microarray.

Fig. S3. Sequence differences in 22 EHEC strains compared with the reference FimH_K514_ or FimH_J96_.

This material is available as part of the online article from http://www.blackwell-synergy.com
