# Supplementary material for: The affinity of the FimH fimbrial adhesin is receptor-driven and quasi-independent of Escherichia coli pathotypes
Source: Mol Microbiol. 2006 Aug 23;61(6):1556–68. doi: 10.1111/j.1365-2958.2006.05352.x (PMC1618777; doi:10.1111/j.1365-2958.2006.05352.x)
Supplement: Fig. S1. — High-mannose microarray. [file mmi0061-1556-FigsS1-S3.pdf]

The affinity of the FimH fimbrial adhesin is receptor driven and quasi-independent of *Escherichia coli* pathotypes

Bouckaert *et al.*

SUPPORTING INFORMATION

Figure S1

High-mannose microarray

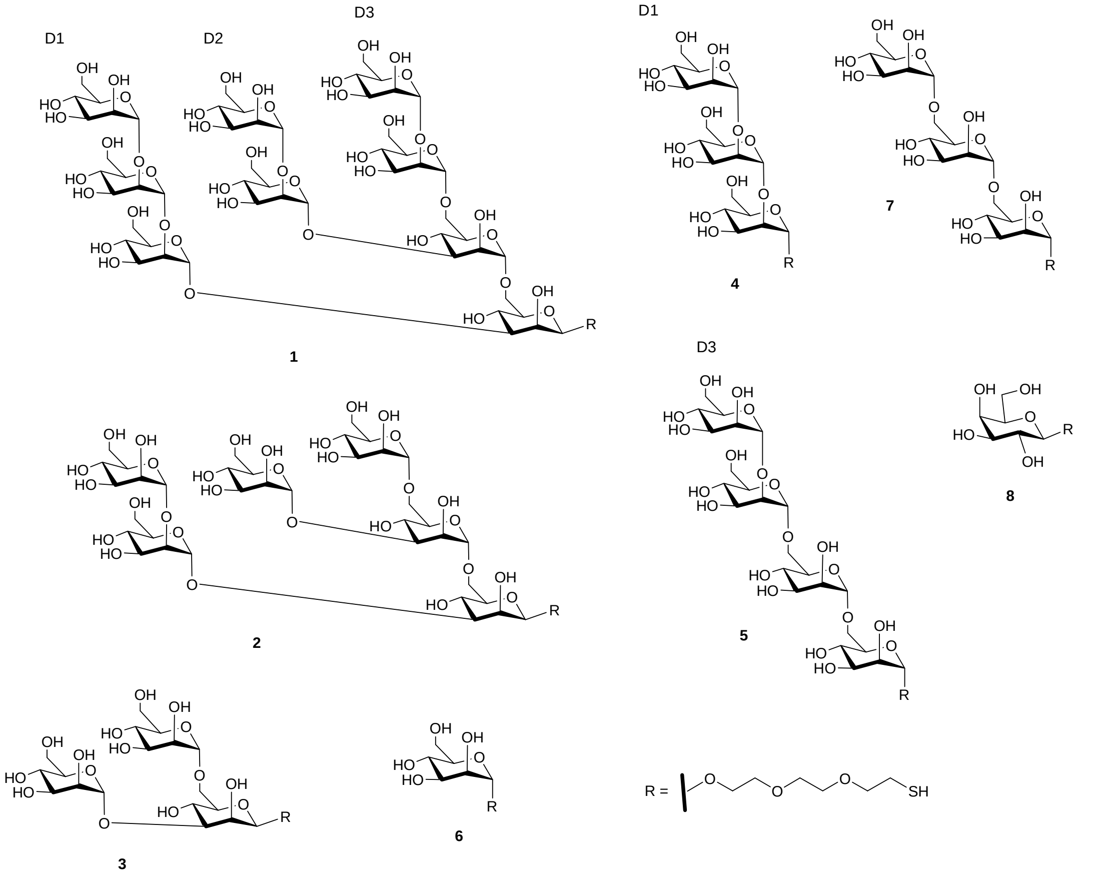

The high mannose microarray was prepared as follows. High-density maleimide slides were prepared from amine-coated GAPS slides (Corning). The slides were incubated overnight at room temperature

in a N,N-dimethylformamide (DMF) solution containing N-succinimidyl 6-maleimidocaproate (1.8 mM) and N,N-diisopropylethylamine (100 mM). Then, the slides were washed 4 times with methyl alcohol, dried under a stream of argon and stored in a dessicator prior to use. High mannose oligosaccharides with a thiol-terminated triethylene glycol linker on their reducing ends were synthesized as previously described (Ratner *et al.* (2002) *Eur J Org Chem* **2002:** 826-833; Ratner *et al.* (2004) *Chembiochem* **5:** 379-382). Thiol containing oligosaccharides were treated with tris(carboxyethyl)phosphine hydrochloride (TCEP, 1 equivalent) in PBS buffer (10 mM, pH 7.4) for 1 h at room temperature and printed on the maleimide glass slides by using a standard DNA array printer (Perkin Elmer). Four different sugar concentrations were employed (2 mM, 1 mM, 0.5 mM, and 0.25 mM) and the resulting spots had an average diameter of 200 μm. Thereafter, the slides were incubated in a humid chamber for 12 h at room temperature, washed twice with distilled water and incubated for 1 h in a solution of 2-mercaptoethanol (1 mM) in PBS buffer (10 mM) to quench all remaining maleimide groups. The slides were washed three times with distilled water, twice with ethanol (95%) and stored in a dry box until use.

Figure S2

Fluorescence response for FimH binding to the carbohydrates linked on the high-mannose microarray.

For detection of bound FimH, we used Penta-His Alexa Fluor 555 antibody, directly binding to the 6-histidine tag of $FimH_{J96}$. This gave higher signal-to-noise ratios than incubation with rabbit anti-FimH followed by hybridization with AlexaFluor-546-labelled anti-rabbit IgG as secondary antibody.

1 oligomannose 9 minus $(GlcNAc)_2$

2 oligomannose 6 minus $(GlcNAc)_2$

3 Manα1–6(Manα1–3)Manβ

4 Manα1–2Manα1–2Manα

5 Manα1–2Manα1–6Manα1–6Manα

6 α–D–mannose

7 Manα1–6Manα1–6Manα

8 β-D-galactose

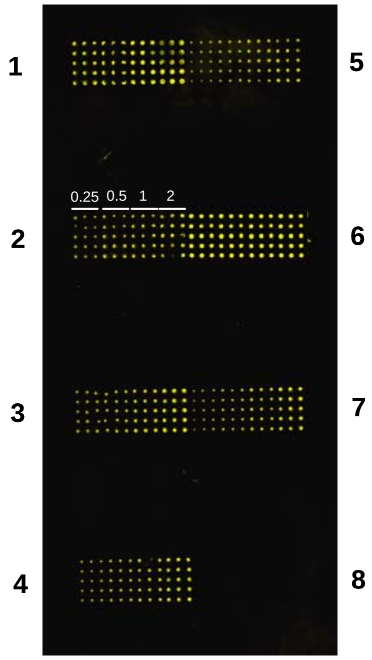

Figure S3

Sequence differences in 22 EHEC strains compared to the reference FimH$_{K514}$ or FimH$_{J96}$

The strains EH10, EH12, EH255, EH297, EH315, EH327, EH330, EH337, EH345, EH349, EH367, EH387, EH406, EH407, EH419, EH422, EH505, EH517 are human clinical isolates. The strains XH182 and XH193 are from Scottish outbreaks (De Baets *et al.* (2004) *Appl Environ Microbiol* **70:** 6309–6314). The strains EH485 and EH514 were isolated from a bovine carcass and from a cow, respectively.

	GenBank acc. n°	2	27	37	66	70	78	82	135	144	223
K514		a	v	d	g	n	s	y	n	f	a
J96		a	v	d	g	n	s	y	n	f	a
EDL933		a	**a**	d	g	n	s	y	**k**	f	a
EH10 (O172:H^{-})	DQ465478	a	**a**	d	g	n	s	y	n	f	a
EH12 (O2:K1:H6)	DQ465479	a	**a**	**h**	**d**	**s**	**n**	y	n	f	a
EH255 (O103:H2)	DQ465480	a	**a**	d	g	n	s	y	n	f	a
EH297 (O157:H7)	DQ465482	a	**a**	d	g	n	s	y	**k**	f	a
EH315 (O145:H^{-})	DQ465481	**t**	**a**	d	g	n	s	y	n	f	a
EH327 (O172:H^{-})	DQ465477	**t**	**a**	d	g	n	s	y	n	f	a
EH330 (O157:H^{-})	DG465490	a	**a**	d	g	n	s	y	n	f	a
EH337 (O121:H^{-})	DQ465483	a	**a**	d	g	n	s	y	n	f	a
EH345 (O157:H7)	DQ465491	a	**a**	d	g	n	s	y	n	f	a
EH349 (O157:H7)	DQ465492	a	**a**	d	g	n	s	y	n	**l**	a
EH367 (O111:H^{-})	DQ465484	a	**a**	d	g	n	s	y	n	f	a
EH387 (O157:H7)	DQ465494	a	**a**	d	g	n	s	y	**k**	f	a
EH406 (O103:H2)	DQ465485	a	**a**	d	g	n	s	y	n	f	a
EH407 (O157:H^{-})	DQ465495	a	**a**	d	g	n	s	y	**k**	f	a
EH419 (O111ac:H^{-})	DQ465486	a	**a**	d	g	n	s	y	n	f	a
EH422 (O26:H^{-})	DQ465487	a	**a**	d	**s**	n	s	**d**	n	f	**v**
EH485 (O157:H7)	DQ465493	a	**a**	d	g	n	s	y	n	f	a
EH505 (O121:H^{-})	DQ465488	a	**a**	d	g	n	s	y	n	f	a
EH514 (O157:H^{-})	DQ465489	a	**a**	d	g	n	s	y	**k**	f	a
EH517 (O157:H7)	DQ465497	a	**a**	d	g	n	s	y	n	f	a
XH182 (O157)	DQ465498	a	**a**	d	g	n	s	y	**k**	f	a
XH193 (O157)	DQ465496	a	**a**	d	g	n	s	y	**k**	f	a
